# Association between modes of delivery and postpartum dietary patterns: A cross-sectional study in Northwest China

**DOI:** 10.3389/fnut.2022.985941

**Published:** 2022-11-10

**Authors:** Lingxi Zhao, Lianlian Wang, Wenling Wang, Zumin Shi, Yuzhao Zhu, Shengping Li, Tiankun Wang, Yu Su, Zhourong Li, Yaqi Wen, Laixi Zhang, Qi Xu, Manoj Sharma, Yong Zhao

**Affiliations:** ^1^College of Public Health, Chongqing Medical University, Chongqing, China; ^2^Research Center for Medicine and Social Development, Chongqing Medical University, Chongqing, China; ^3^Research Center for Public Health Security, Chongqing Medical University, Chongqing, China; ^4^Department of Obstetrics, The First Affiliated Hospital of Chongqing Medical University, Chongqing, China; ^5^Perinatology Center, Gansu Provincial Maternity and Child-Care Hospital, Lanzhou, China; ^6^Department of Human Nutrition, College of Health Sciences, QU Health, Qatar University, Doha, Qatar; ^7^Department of Environmental and Occupational Health, School of Public Health, University of Nevada, Las Vegas, NV, United States; ^8^Chongqing Key Laboratory of Child Nutrition and Health, Children’s Hospital of Chongqing Medical University, Chongqing, China

**Keywords:** parturient, dietary pattern, cesarean delivery, dietary behavior, postpartum period

## Abstract

**Objective:**

Puerperae’ dietary patterns (DPs) during the puerperium may be influenced by the mode of delivery, but population studies on this topic are scarce. This study aims to explore the relationship between DPs and different modes of delivery among puerperae.

**Methods:**

A cross-sectional study was conducted on 3,345 parturients in Lanzhou, China. The postpartum food intake was measured by a food frequency questionnaire (FFQ). Factor analysis was used to determine the DPs. Multiple linear regression was employed to examine the association between the mode of delivery and DP.

**Results:**

In this study, two DPs, i.e., traditional and modern DPs, were identified. Traditional DP was characterized by high energy-adjusted intake of tubers, coarse cereals, rice, whole grains, fishery products, and eggs. Modern DP included a high intake of coffee, non-sugary drinks, wine, tea, and fishery products. Compared with participants with vaginal delivery (reference category), cesarean section had an inverse association with modern DP (β: −0.11, 95% CI: −0.36, −0.09). A significant interaction was found between education level, monthly household income, alcohol drinking, and modes of delivery. The inverse association between cesarean section and modern DP or the intake of coffee was significant among puerperae with higher or lower monthly household income. However, the inverse association between cesarean section and traditional DP was only found among puerperae with higher monthly household income. Moreover, among the participants with high education, cesarean section was positively associated with intake of vegetables.

**Conclusion:**

Cesarean puerperae with higher levels of education and those with lower and higher monthly household income had less unhealthy foods intake than those who had vaginal delivery. They need to be accounted for in educational programs and interventions focused on healthy diet recommendations in puerperium.

## Introduction

Diet during the peripartum period is important to the health of mother and child (1) and provides an opportunity to change their old unhealthy eating habits (2). Dietary patterns (DPs), rather than individual food groups, foods, or nutrients, provide a more comprehensive approach to assess both health and environmental outcomes related to the diet (3). A high-quality diet, such as the healthy eating pattern of the Mediterranean diet, is internationally recommended for pregnant and breastfeeding women (4). Nevertheless, several studies on a national or international scale showed that pregnant women do not generally follow the recommended healthy diet (5–11), which can contribute to the suboptimal intake of key nutrients essential to the health of mother and child (1).

Evidence suggested that food intake patterns may be influenced by the physiological and behavioral demands of pregnancy and postpartum (2, 12–15). The mode of delivery is an important factor that can influence a woman’s postnatal behavior and quality of life owing to different painful conditions. Compared with women who have vaginal delivery, the risk of reduction in health and wellbeing during postpartum period is higher among women who had a cesarean section (16). Moreover, Chinese cesarean delivery rate in China is highest in the world (17, 18). Therefore, many studies focus on the postnatal nutritional care of cesarean puerperae (19). Some studies found that women who had a cesarean section are offered oral fluids and food. Moreover, some studies determined that coffee can be given to patients to enhance the recovery of their gastrointestinal function after elective cesarean section (20, 21). However, studies that investigated the association between vaginal delivery and postpartum diet are still scarce.

A limited number of studies have focused on the postnatal dietary patterns (DPs) (21, 22) in parturients with different delivery modes. It is unknown to what degree delivery modes affect postnatal DPs in China. Therefore, this study aims to examine the relationship between DPs and mode of delivery of puerperae to provide a scientific basis for health promotion and interventions focused on healthy diet recommendations in puerperium (22, 23).

## Materials and methods

### Study design and sample collection

This study is a cross-sectional survey conducted at the maternity outpatient clinic of Lanzhou Maternity and Child Health Hospital in Gansu Province from October 2019 to April 2021. A convenience sampling method was used in this study. A questionnaire survey was conducted by the hospital’s obstetrician and gynecologist on women who underwent puerperal examinations at the hospital. This study was conducted in accordance with the Declaration of Helsinki, and the protocol was approved by the Ethics Committee of Chongqing Medical University (record number 2018-131). All participants provided their written informed consent.

Literature demonstrated that the cesarean section rate in China is approximately 36.7% (24). According to the formula of sample size calculation,


$$N=\left(Z_{\alpha }^{2}\times p\times q\right)/d^{2}$$

*p* is 0.367. *q* = 1 − *p* = 0.633. The margin of error (*d*) = 0.10 × *p* = 0.0367, and α is 95%. The calculated sample size was 2,703, considering the possibility of 15% non-response rate. The minimum sample size needed for this study was calculated to be 3,180.

**FIGURE 1:**
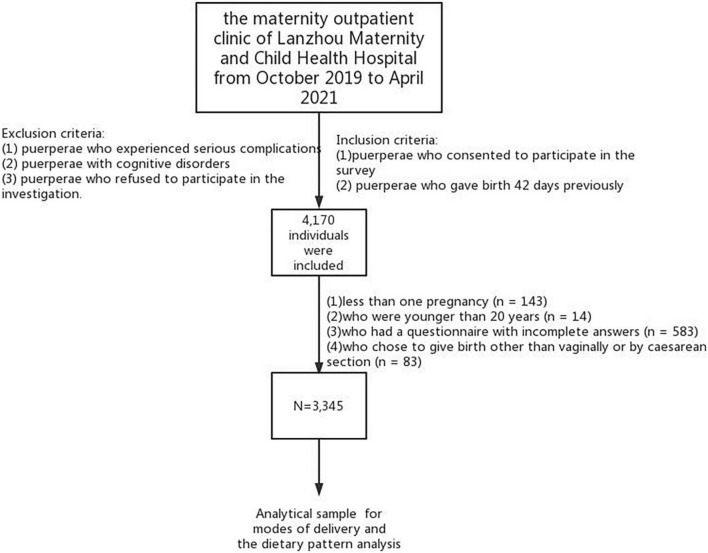
Participant flow chart.

Puerperae who (1) consented to participate in the survey and (2) gave birth 42 days previously were included in this study. Exclusion criteria were as follows: puerperae (1) who experienced serious complications, (2) with cognitive disorders and (3) who refused to participate in the investigation. A total of 3,345 responses were included in the analysis after the subjects with less than one pregnancy (*n* = 143), who were younger than 20 years (*n* = 14), who had a questionnaire with incomplete answers (*n* = 583) and who chose to give birth other than vaginally or by cesarean section (*n* = 83) were excluded (Figure 1). Ultimately, 3,345 participants were enrolled.

### Dietary assessment and food grouping

A qualitative food frequency questionnaire (FFQ) was used to assess the dietary intake of puerperae participating in this study on their 42nd day after delivery. Of all the dietary assessment methods: 24-h recall, food record, food history, and FFQ, the last is a reliable and inexpensive data collection method, which has been widely allows the identification and evaluation of food patterns in epidemiological studies and can assess diet quality (25). All the foods included in the current analysis were selected according to the Dietary Guidelines for Chinese lactating women and common postpartum practices reported by previous studies (26). The final version was modified by epidemiologists, statisticians, multidisciplinary experts in nutrition, and a health management professor to ensure the content of the questionnaire was valid and had used in previous studies (26–28). A total of 78 foods items were included in the FFQ. In the analysis the food items were categorized into 21 food groups based on the similarity of nutrition profiles and cooking method. The 21 food groups were vegetables, fruits, freshwater fish, seafood, red meat, poultry, processed meat, milk, dairy desserts, eggs, sugary drinks, non-sugary drinks, tea, coffee, wine, rice, wheat, coarse cereals, potatoes, whole grains and cakes and confectionery. We did not collect data on the amount consumed in the FFQ, thus we calculated only the daily consumption frequency for each food group. Although the FFQ has not been validated, the food items included in the FFQ were similar to other validated FFQ in China (29). All participants were asked to record their habitual intake frequency for each food group within the last year according to the following categories:

1– Less than once a month
2– Once a month
3– Two to three times a month
4– Once a week
5– Two to three times a week
6– Four to six times a week
7– Once a day
8– Twice a day
9– Three to four times a day
10– More than five times a day

For the presented analyses, the information on food consumption frequency was transformed into the number of times each food was consumed over a week. These food groups are listed in Supplementary Table 7.

### Mode of delivery

The questionnaire categorized the mode of delivery into five types (cesarean delivery, normal delivery, delivery by forceps, breech delivery, and vacuum extraction). Given that the number of cesarean and normal deliveries accounted for 97.36% of the total number of deliveries, those who chose other delivery methods were excluded. Finally, 2,187 cesarean and 1,158 vaginal puerperae were included in the analysis.

### Covariates

The sociodemographic characteristics and health-related lifestyles were also obtained through the questionnaire. The sociodemographic variables were as follows: age (20–34 years/35 years or above), ethnicity (Han/minority) and residence (urban/rural). Marital status was recorded as primary marriage or other (remarried, divorced, or other). Educational level was recorded as two categories: secondary education or below (senior high school or below) and tertiary education (university or above). The average monthly household income was categorized as <¥4,500, ¥4,500–¥9,000 or >¥9,000 (30). Dietary caregivers were divided into three categories, including self/husband, in-laws/parents and maternity matron/nanny. The prepregnancy body mass index (BMI) was calculated on the basis of the self-reported preconception weight. BMI was rated as follows: <18.5 kg/m^2^, thin; 18.5–24.9 kg/m^2^, normal; 25.0–29.9 kg/m^2^, overweight; and 30.0–40.0 kg/m^2^, obese (31). Suffering from a postpartum disease and alcohol drinking were classified into two categories, i.e., yes or no. Singleton or twin pregnancies was classified into two categories, i.e., singleton pregnancies or twin pregnancies. Smoking status was divided into three categories, including current smoker, ex-smoker, and non-smoker.

### Statistical analysis

All statistical analyses were performed using STATA (version 17, StataCorp, College Station, TX, USA) and the software GraphPad Prism 8. Demographic characteristics were described using frequencies and percentiles, which were all categorical variables. Chi-square test was used to compare the distributions of the delivery modes in lower versus higher DPs categories (split at the median of DP scores). DPs’ Chi-square and multiple linear regression were utilized for categorical variables and continuous variables, respectively. Differences between the mode of delivery and demographic characteristic variables were analyzed using chi-square tests. The intake of the 21 food groups was included in the factor analysis (32). A factor analysis (principal component extraction with varimax rotation) of specific items revealed four factors (with eigenvalue >1), explaining 64.0% of the total variance. Finally, two DPs were determined in accordance with factor interpretability, which explained 52.6% of the total variance. Food groups with component loading ≥| 0.30| are shown in Table 1. The factors were rotated with varimax to improve the interpretability and minimize the correlation between the factors. Participants were assigned a pattern-specific factor score, which was calculated as the sum of the product of the factor loading coefficients and standardized daily intake of each food associated with the pattern. Factor loadings were included in the calculation of pattern scores.

**TABLE 1:** Component loadings for dietary patterns.

	Component 1^a^	Component 2^b^
Vegetables	0.553	
Fruits	0.586	
Poultry	0.554	0.342
Fishery products	0.512	0.43
Processed meat products	0.511	0.467
Fish	0.501	0.426
Red meat	0.441	0.394
Dairy desserts	0.593	0.339
Milk	0.563	0.322
Tubers	0.691	
Coarse cereals	0.677	
Rice	0.668	
Whole grains	0.645	0.356
Eggs	0.625	
Wheat	0.616	
Cakes	0.57	0.465
Coffee		0.933
Non-sugary drinks		0.888
Wine		0.871
Sugary drinks		0.865
Tea		0.844

Component loadings for dietary patterns derived from qualitative food frequency questionnaire data completed by women who underwent postnatal check-ups.

^a^Component 1, called traditional dietary pattern, is characterized by high energy-adjusted intake of staple food (tubers, coarse cereals, rice, and whole grains), eggs, meat, fish and fishery products, fruits, and vegetables.

^b^Component 2, called modern dietary pattern, is characterized by high energy-adjusted intake of coffee, tea, drinks, and wine and low intake of meat and whole grains.

Multiple linear regression was conducted to verify the association between the variables describing the mode of delivery (independent variables) and dietary intake pattern (dependent variables). A set of multiple linear regression models was built: Model 1 – adjusting the age groups; Model 2 – further adjusting the residence, education, income, and dietary caregivers and Model 3 – further adjusting the prepregnancy BMI and suffering from a postpartum disease based on Model 2.

The multiplicative interaction between dietary intake and demographic characteristics (i.e., marital status, parity, and education level) was tested by adding the product of the variables to the multivariable model.

## Results

### Characteristics of study participants

A total of 3,345 puerperal women were included in this survey. About 82.2% of puerperae were aged 20–34 years. In the sample, 69.6% of the participants were first birth, 82.8% obtained tertiary education, 62.5% had been taken into care by parents or parents-in-law, and 25.2% had a monthly household income of ≤¥4,500. Statistically significant modes of delivery differences in demographic characteristics were found in parity, marital status, education level, residence, and age. Per IOTF (international obesity task force) standards, prepregnancy BMI categories significantly differed (*p* < 0.001). Modes of delivery differences were also found in dietary caregivers, singleton or twin pregnancies and suffering from a postpartum disease (*p* < 0.001, Table 2).

**TABLE 2:** Basic demographic characteristics.

Factor	Total *N* = 3,345	Delivery mode		*P*-value
		Vaginal delivery *N* = 1,158	Cesarean section *N* = 2,187	
Parity				0.002*
First birth	2,327 (69.6%)	766 (66.1%)	1,561 (71.4%)	
Second birth	1,018 (30.4%)	392 (33.9%)	626 (28.6%)	
Ethnicity				0.075
Han	3,006 (92.7%)	1,025 (91.6%)	1,981 (93.3%)	
Minority	236 (7.3%)	94 (8.4%)	142 (6.7%)	
Marital status				<0.001*
Primary marriage	3,213 (96.7%)	1,088 (94.9%)	2,125 (97.6%)	
Other marital status	110 (3.3%)	58 (5.1%)	52 (2.4%)	
Educational level				<0.001*
Secondary education and below	573 (17.2%)	254 (22.0%)	319 (14.6%)	
Tertiary education	2,765 (82.8%)	902 (78.0%)	1,863 (85.4%)	
Residence				<0.001*
Urban	3,008 (90.9%)	993 (87.3%)	2,015 (92.9%)	
Rural	300 (9.1%)	145 (12.7%)	155 (7.1%)	
Age				<0.001*
20–34	2,749 (82.2%)	876 (75.6%)	1,873 (85.6%)	
≥35	596 (17.8%)	282 (24.4%)	314 (14.4%)	
Dietary caregivers				0.032*
Themselves/husband	472 (15.2%)	187 (17.4%)	285 (14.0%)	
Parents/parents-in-law	1,944 (62.5%)	663 (61.7%)	1,281 (63.0%)	
Nanny/maternity matron	693 (22.3%)	225 (20.9%)	468 (23.0%)	
Monthly household income				0.076
≤¥4,500	853 (25.5%)	313 (27.0%)	540 (24.7%)	
¥4,501–9,000	1,181 (35.3%)	380 (32.8%)	801 (36.6%)	
>¥9,000	1,311 (39.2%)	465 (40.2%)	846 (38.7%)	
Pre-pregnancy BMI				<0.001*
Thin	503 (15.0%)	131 (11.3%)	372 (17.0%)	
Normal	1,993 (59.6%)	671 (57.9%)	1,322 (60.4%)	
Overweight	393 (11.7%)	179 (15.5%)	214 (9.8%)	
Obesity	456 (13.6%)	177 (15.3%)	279 (12.8%)	
Suffering from postpartum disease				0.035*
No	1,184 (73.5%)	436 (76.6%)	748 (71.8%)	
Yes	427 (26.5%)	133 (23.4%)	294 (28.2%)	
Singleton or twin pregnancies				<0.001
Singleton pregnancies	3,243 (97.3%)	1,068 (92.7%)	2,175 (99.7%)	
Twin pregnancies	90 (2.7%)	84 (7.3%)	6 (0.3%)	
Smoking				0.57
Current smoker	38 (1.1%)	16 (1.4%)	22 (1.0%)	
Ex-smoker	47 (1.4%)	15 (1.3%)	32 (1.5%)	
Non-smoker	3,237 (97.4%)	1,116 (97.3%)	2,121 (97.5%)	
Alcohol drinking				0.71
No	2,450 (78.2%)	847 (78.6%)	1,603 (78.0%)	
Yes	682 (21.8%)	231 (21.4%)	451 (22.0%)	

*P < 0.05.

### Dietary patterns

Two DPs, namely, traditional and modern DPs, were identified. Food with loading ≥ 0.3 on traditional DP included a high energy-adjusted intake of tubers (loading = 0.691), coarse cereals (loading = 0.677), rice (loading = 0.668), whole grains (loading = 0.645), fishery products (loading = 0.512), and eggs (loading = 0.625). Foods loaded on modern DP included a low intake of processed meat products (loading = 0.467) and cakes (loading = 0.465) and a high intake of coffee (loading = 0.933), non-sugary drinks (loading = 0.888), wine (loading = 0.871), tea (loading = 0.844), and fishery products (loading = 0.43). The food groups with a component loading ≥| 0.30| are shown in Table 1.

### Association between mode of delivery and postpartum dietary intake

**FIGURE 2:**
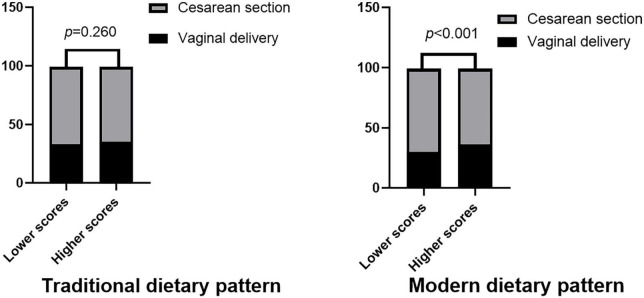
Associations between modes of delivery and postpartum dietary intake.

The delivery mode was associated with the modern DPs, such that vaginal puerperae were more likely to be in the upper half of the modern DPs compared to cesarean puerperae (Figure 2). The multiple linear regression results, including the β-coefficients (95% CI) between the delivery modes and dietary intake scores, are presented in Table 3. After the adjustment of the sociodemographic variables and health-related behaviors, compared with vaginal delivery (reference category), cesarean puerperae had an inverse association with modern DP (β:−0.11, 95% CI: −0.36, −0.09), indicating that the woman who delivered by cesarean section had low adherence to modern DP. However, no difference was observed in the dietary scores between the different delivery modes and traditional DP.

**TABLE 3:** Associations between modes of delivery and postpartum dietary intake.

	Model 1^a^		Model 2^b^		Model 3^c^	
	β (95% CI)	*P*-value	β (95% CI)	*P*-value	β (95% CI)	*P*-value
Traditional dietary pattern						
Vaginal delivery (Ref)						
Cesarean section	−0.02 (−0.13, 0.06)	0.629	0.01 (−0.11, 0.07)	0.762	−0.02 (−0.05, 0.07)	0.477
Modern dietary pattern						
Vaginal delivery (Ref)						
Cesarean section	−0.07 (−0.24, −0.07)	<0.001*	−0.06 (−0.16, −0.08)	0.006*	−0.11 (−0.36, −0.09)	0.001*

^a^Model 1 adjusted for age.

^b^Model 2 further adjusted for residence, education, income and dietary caregivers.

^c^Model 3 further adjusted for pre-pregnancy BMI, suffering from postpartum diseases, singleton or twin pregnancies, monthly household income, smoking, and alcohol drinking.

*P < 0.05.

### Subgroup analyses of the association between mode of delivery and postpartum dietary intake

The mode of delivery had no interaction with marital status, age, singleton, or twin pregnancies and smoking in relation to association with traditional DP, modern DP and the intake of vegetable, fruit, and coffee. However, we observed significantly inverse effects of monthly household income on the association between the cesarean section and traditional DP (*p* = 0.025) as well as modern DP (*p* = 0.036).

An inverse association between modes of delivery and the traditional DP was only found among puerperae with monthly household income >¥9,000. However, the inverse association between modes of delivery and the modern DP was observed among puerperae with monthly household income ≤¥4,500 or > ¥9,000 and non-drinking. Furthermore, a positively association between cesarean section and the intake of vegetable was found among tertiary education level. And, the observed inverse association between modes of delivery and the intake of coffee remained statistically significant among participants with monthly household income ≤¥4,500 or >¥9,000 (Tables 4, 5 and Supplementary Table 1).

**TABLE 4:** Subgroup analyses of associations between modes of delivery and postpartum dietary intake^#^.

Factor	Traditional dietary pattern	*P* ^1^	Modern dietary pattern	*P* ^1^	Vegetable	*P* ^1^
Marital status						
Primary marriage	−0.05 (−0.14, 0.05)	0.574	−0.16 (−0.27, −0.06)*	0.327	0.54 (−0.13, 1.22)	0.377
Other marital status	0.18 (−0.35, 0.72)		−0.13 (−0.35, 0.08)		2.47 (−1.70, 6.64)	
Age						
20–34	−0.04 (−0.14, 0.07)	0.713	−0.15 (−0.25, −0.06)*	0.716	0.52 (−0.22, 1.25)	0.559
≥35	−0.05 (−0.26, 0.17)		−0.50 (−1.11, 0.11)		0.99 (−0.56, 2.54)	
Education level						
Secondary education and below	−0.03 (−0.23, 0.17)	0.875	−0.14 (−0.41, 0.13)	0.790	−1.00 (−2.60, 0.60)	0.049
Tertiary education	−0.02 (−0.13, 0.08)		−0.14 (−0.24, −0.05)*		0.73 (0.00, 1.46)*	
Singleton or twin pregnancies						
Singleton pregnancies	−0.04 (−0.13, 0.06)	0.961	−0.15 (−0.25, −0.06)*	0.198	0.62 (−0.04, 1.29)	0.778
Twin pregnancies	−0.14 (−1.45, 1.17)		−0.96 (−2.36, 0.43)		0.97 (−8.85,10.80)	
Monthly household income						
≤4,500	−0.06 (−0.25, 0.12)	0.025	−0.29 (−0.51, −0.07)*	0.036	0.72 (−0.64, 2.08)	0.983
4,501–9,000	0.13 (−0.03, 0.29)		0.01 (−0.14, 0.15)		0.23 (−0.88, 1.35)	
>9,000	−0.18 (−0.33, −0.03)*		−0.22 (−0.36, −0.09)*		0.67 (−0.38, 1.72)	
Smoking						
Current smoker	−0.40 (−1.80, 1.00)	0.827	0.49 (−0.39, 1.36)	0.099	−3.33 (−12.66, 6.00)	0.555
Ex-smoker	0.07 (−2.41, 2.54)		−0.24 (−2.87, 2.40)		−1.32 (−10.58, 7.94)	
Non-smoker	−0.04 (−0.14, 0.05)		−0.16 (−0.25, −0.07)*		0.65 (−0.02, 1.32)	
Alcohol drinking						
No	−0.08 (−0.19, 0.03)	0.130	−0.24 (−0.34, −0.13)*	0.005	0.42 (−0.33, 1.18)	0.221
Yes	0.09 (−0.11, 0.29)		0.04 (−0.14, 0.22)		1.13 (−0.28, 2.54)	

^1^*P* for interaction.

*P < 0.05.

^#^Vaginal delivery as the reference group.

**TABLE 5:** Subgroup analyses of associations between modes of delivery and postpartum dietary intake in the total sample^#^.

Factor	Fruits	*P* ^1^	Coffee	*P* ^1^
Marital status				
Primary marriage	−0.06 (−0.77 to 0.65)	0.139	−1.63 (−3.04 to −0.22)*	0.233
Other marital status	2.87 (−2.00 to 7.73)		3.93 (−3.33 to 11.19)	
Age				
20–34	−0.11 (−0.88 to 0.67)	0.145	−1.69 (−3.25 to −0.14)*	0.483
≥35	1.11 (−0.58 to 2.80)		−0.34 (−3.42 to 2.74)	
Education level				
Secondary education and below	−1.51 (−3.67 to 0.65)	0.005	−2.05 (−5.03 to 0.93)	0.372
Tertiary education	0.50 (−0.23 to 1.23)		−0.89 (−2.43 to 0.65)	
Singleton or twin pregnancies				
Singleton pregnancies	0.14 (−0.57 to 0.84)	0.507	−1.31 (−2.70 to 0.08)	0.209
Twin pregnancies	−2.22 (−12.27 to 7.83)		−13.18 (−29.81 to 3.45)	
Monthly household income				
≤4,500	−0.54 (−2.13 to 1.05)	0.208	−2.74 (−5.44 to −0.03)*	0.020
4,501–9,000	0.72 (−0.42 to 1.85)		1.44 (−0.95 to 3.83)	
>9,000	−0.12 (−1.19 to 0.95)		−3.12 (−5.31 to −0.93)*	
Smoking				
Current smoker	−4.05 (−14.22 to 6.13)	0.906	−7.64 (−23.19 to 7.92)	0.907
Ex-smoker	2.02 (−7.97 to 12.00)		−2.47 (−27.16 to 22.22)	
Non-smoker	0.11 (−0.60 to 0.82)		−1.42 (−2.82 to −0.02)*	
Alcohol drinking				
No	−0.10 (−0.90 to 0.71)	0.139	−1.70 (−3.25 to −0.14)*	0.548
Yes	0.53 (−0.91 to 1.96)		−0.60 (−3.64 to 2.44)	

^1^*P* for interaction.

*P < 0.05.

^#^Vaginal delivery as the reference group.

## Discussion

This study analyzed DPs in a sample of parturients in Lanzhou Maternal and Child Health Hospital on their 42nd day after childbirth and the association among mode of delivery, DP and food intake. In this study, two DPs (e.g., traditional and modern DPs) were identified. This study also found that modes of delivery had an inverse association with modern DP. Furthermore, the modern DP score and the intake of coffee were inversely associated with modes of delivery among puerperae under 35 years of age. Among the participants with secondary education or lower, modes of delivery had an inverse association with intake of vegetables.

Our results were in accordance with the literature. Although some investigators advocated the adoption of a high-quality DP during the postnatal period, such as the Mediterranean diet model or healthy food pyramid (33), many maternal diets are unreasonable (1, 34–39). Two DPs were identified in this study. Specifically, the traditional DP, which was a high-quality DP, was characterized primarily by high consumption of rice, vegetables, fishery products, whole grains, dairy desserts, poultry, and fish. However, the modern DP was a low-quality DP involving the consumption of a combination of wheat, coffee, sugary drinks, wine, cakes, and processed meat products. Thus, there is a gap between this DP and the diet recommended by Dietary Guidelines for Chinese lactating women (40). The nutritional status of a woman during lactation is not only critical for her health, but for future generations (41). Furthermore, the puerperium diet of Chinese women is determined by the culture of “zuo yuezi,” some traditional eating behaviors the one month postpartum. During this period, their behavior in relation to diet, activity and hygiene is determined by the theory of traditional Chinese medicine (TCM). Several “zuo yuezi” practices are beneficial, including eating more, eating protein rich food. However, its recommendation to avoid cold foods (e.g., vegetables and fruits) is not consistent with the Dietary Guidelines for Chinese lactating women (42). In brief, the attention and interventions of the health team should continue into postpartum so that the adoption of healthy eating habits is guaranteed (43).

Various factors may affect the choice of postpartum DP (e.g., age, gender, and economic level) (30, 33), but the effect of mode of delivery on postnatal DP has attracted limited attention. This study found no association between traditional DP and delivery mode. However, compared with vaginal delivery, cesarean puerperae had an inverse association with modern DP. Compared with normal vaginal delivery, cesarean section was inversely associated with postpartum diseases, causing healthcare to pay attention to postoperative care in cesarean delivery (44). The postnatal health and wellbeing of woman who delivered by vaginal section is higher than those of woman who delivered by cesarean section (45). During the postpartum period, women who delivered by vaginal section spend more time on childcare, preparing foods for family and cannot take too much time on maintaining healthy diet (2, 46, 47).

This study determined that the intake of vegetables, fruits and coffee was correlated with cesarean section only when adjusting for age. Different from complications after vaginal delivery, postoperative ileus (POI) is a frequent occurrence after a cesarean section. Increased attention should be paid to the postpartum diet of cesarean puerperae to promote the return of the gastrointestinal function (48). The guidelines for postoperative cesarean section recommend a high intake of fruits and vegetables to prevent constipation (44). Moreover, a review published in 2021 showed that postoperative coffee consumption can likely reduce POI incidence after a cesarean section (20). Nevertheless, no correlation was observed in this study between the consumption of fruits, vegetables or coffee and delivery mode after the other confounding factors, including residence, education, income, and dietary caregivers, were controlled. This relationship was not from a causal effect and might be attributed to confounding factors.

In the subgroup analyses, the interaction between monthly household income and modes of delivery to traditional/modern DPs is interesting. The significantly inverse interaction was found between monthly household income and modern DPs among puerperae with higher or lower monthly household income. This finding was consistent with a qualitative research which thought that the diet of Chinese puerperae is susceptible to traditional beliefs regardless of income (42). In addition, this interaction also found among non-drinking cesarean puerperae. This suggests that daily drinking habits can influence the choices of modern DPs for cesarean puerperae. However, the inverse association between cesarean section and traditional DP was only found among puerperae with higher monthly household income. The possible cause is that Chinese traditional beliefs and practices believe that puerperae should eat more, eat protein rich food and avoid “cold” food (e.g., fruits and vegetables) (42). In this study, the cesarean puerperae with a high level of education were likely to increase the intake of vegetables, and this finding was consistent with the findings of a Spanish cross-sectional study (33, 49). Therefore, education on postpartum diet health knowledge for parturients with different levels of education and household income and their dietary caregivers should be strengthened.

Some limitations of this study should be addressed. Firstly, due to the cross-sectional study design it was not possible to claim the causal relationship between modes of delivery and DPs; therefore, prospective studies are needed to confirm the findings of this study. Secondly, food consumption data are from a qualitative FFQ, and two individuals with an identical consumption frequency may have different true consumptions of a particular food owing to differences in the consumed portion sizes (50, 51), this may have reduced the ability to assess the diet quality by FFQs. This loss of detail is inherent in qualitative FFQs. Thirdly, questionnaires had been completed by the medical staff may led to interviewer bias. Fourthly, the data of elective and emergency cesarean sections and pregnancy diet should be considered. Fifthly, it was difficult for us to conduct a follow-up study because of the COVID-19 pandemic and the fact that the investigators were the hospital’s obstetrician and gynecologist, so the reliability of FFQ test was only conducted among a small number of participants. However, Qin et al. (52) demonstrated that portion size adds only limited information to the variance in food intake, suggesting that standard portion size specifications may not introduce a large error in the estimation of food intake. Secondly, the use of a questionnaire is limited owing to the misestimation in the consumption frequency for certain foods when FFQ is used. Thirdly, this study investigated only parturients in the northwest, lacking samples from multicenter surveys. However, obtaining such samples is not logistically and economically possible in this study, considering the number of respondents. Despite research limitations, they do not affect the importance of this study.

## Conclusion

In conclusion, puerperium is a critical period for postpartum recovery. However, modern DP is a low-quality DP. Moreover, this study found that the choice of modern DP during this period is related to the mode of delivery. Furthermore, significant interaction exists among monthly household income, education level, and modes of delivery. Thus, based on our findings health professionals should pay attention to the dietary intake of those with a low literacy and those had normal vaginal delivery with lower and higher monthly household income.

## Data availability statement

The original contributions presented in this study are included in the article/Supplementary material, further inquiries can be directed to the corresponding author.

## Ethics statement

The studies involving human participants were reviewed and approved by the Ethics Committee of Chongqing Medical University. The patients/participants provided their written informed consent to participate in this study.

## Author contributions

LiZ and LW performed the statistical analysis, wrote the manuscript, and was primarily responsible for the final content. ZS, WW, and LaZ were involved in the data extraction and verification. YuZ, YS, SL, TW, and MS provided guidance and suggested revisions. YoZ provided critical updates to the final manuscript. All authors contributed to the article and approved the submitted version.
