# Supplementary material for: Association between modes of delivery and postpartum dietary patterns: A cross-sectional study in Northwest China
Source: Front Nutr. 2022 Nov 10;9:985941. doi: 10.3389/fnut.2022.985941 (PMC9686425; doi:10.3389/fnut.2022.985941)
Supplement: Supplementary file 1 [file Data_Sheet_1.docx]

**Table 1.** Associations between modes of delivery and postnatal intake of vegetables, fruits and coffee.

|  | **Model 1 ^a^** | | **Model 2 ^b^** | | **Model 3 ^c^** | |
| --- | --- | --- | --- | --- | --- | --- |
|  | **β (95% CI)** | ***P*-value** | **β (95% CI)** | ***P*-value** | **β (95% CI)** | ***P*-value** |
| **Vegetable** | | | | | | |
| Vaginal delivery (Ref) | | | | |  |  |
| Cesarean section | -0.05 (-0.16, -1.50) | 0.017 | 0.02 (-0.18, 1.06) | 0.166 | 0.02(-0.52, 1.36) | 0.381 |
| **Fruits** | | | | | | |
| Vaginal delivery (Ref) | | | | |  |  |
| Cesarean section | 0.01 (-0.66, 0.66) | 0.955 | 0.01 (-0.63, 0.71) | 0.907 | -0.02(-1.36, 0.62) | 0.464 |
| **Coffee** | | | | | | |
| Vaginal delivery (Ref) | | | | |  |  |
| Cesarean section | -0.04 (-2.78, -0.21) | 0.023 | -0.03 (-2.32, -0.29) | 0.126 | -0.05(-3.57, 0.32) | 0.101 |

**^a^** Model 1 adjusted for age groups.

**^b^** Model 2 further adjusted for residence, education, income and dietary caregivers.

c Model 3 further adjusted for pre-pregnancy BMI, suffering from postpartum diseases, singleton or twin pregnancies, monthly household income, smoking and alcohol drinking.

**Table 2.** After the adjustment of model 3 the association between modes of delivery and traditional dietary pattern.

| Varibles | β (95% CI) | *P*-value |
| --- | --- | --- |
| **Delivery mode** |  |  |
| Vaginal delivery (Ref) |  |  |
| Cesarean section | -0.02(-0.05, 0.07) | 0.477 |
| **Age** |  |  |
| 20-34(Ref) |  |  |
| ≥35 | 0.02(-0.10, 0.22) | 0.488 |
| **Residence** |  |  |
| Urban(Ref) |  |  |
| Rural | -0.01(-0.27, 0.18) | 0.721 |
| **Educational level** |  |  |
| Secondary education and below(Ref) |  |  |
| Tertiary education | -0.09(-0.41, 0.07) | 0.006 |
| **Monthly Household income** |  |  |
| ≤4500(Ref) |  |  |
| 4501-9000 | -0.09(-0.34, -0.03) | 0.021 |
| >9000 | -0.14(-0.43, -0.12) | <0.001 |
| **Dietary caregivers** |  |  |
| Themselve/husband(Ref) |  |  |
| Parents/Parents-in-law | -0.01(-0.20, 0.160) | 0.801 |
| Nanny/maternity matron | 0.04(-0.11, 0.30) | 0.353 |
| **Pre-pregnancy BMI** |  |  |
| Thin(Ref) |  |  |
| Normal | -0.04(-0.24, 0.09) | 0.389 |
| Overweight | -0.02(-0.30, 0.16) | 0.572 |
| Obesity | -0.06(-0.40, 0.04) | 0.111 |
| **Suffering from postpartum disease** |  |  |
| No(Ref) |  |  |
| Yes | 0.01(-0.12, 0.15) | 0.807 |
| **Singleton or twin pregnancies** |  |  |
| Singleton pregnancies(Ref) |  |  |
| Twin pregnancies | 0.01(-0.33, 0.44) | 0.791 |
| **Smoking** |  |  |
| Current smoker(Ref) |  |  |
| Ex-smoker | -0.07(-1.36, 0.25） | 0.179 |
| Non-smoker | -0.07(-1.11,0.22) | 0.192 |
| **Alcohol drinking** |  |  |
| No(Ref) |  |  |
| Yes | -0.02(-0.20, 0.11) | 0.548 |

**Table 3.** After the adjustment of model 3 the association between modes of delivery and modern dietary pattern.

| Varibles | β (95% CI) | *P*-value |
| --- | --- | --- |
| **Delivery mode** |  |  |
| Vaginal delivery (Ref) |  |  |
| Cesarean section | -0.11(-0.36, -0.09) | 0.001* |
| **Age** |  |  |
| 20-34(Ref) |  |  |
| ≥35 | 0.04(-0.07, 0.26) | 0.249 |
| **Residence** |  |  |
| Urban(Ref) |  |  |
| Rural | 0.10(0.14, 0.60) | 0.001 |
| **Educational level** |  |  |
| Secondary education and below(Ref) |  |  |
| Tertiary education | -0.05(-0.31, 0.04) | 0.130 |
| **Monthly Household income** |  |  |
| ≤4500(Ref) |  |  |
| 4501-9000 | -0.05(-0.27, 0.05) | 0.188 |
| >9000 | -0.07(-0.30, 0.01) | 0.062 |
| **Dietary caregivers** |  |  |
| Themselve/husband(Ref) |  |  |
| Parents/Parents-in-law | 0.01(-0.17, 0.19) | 0.936 |
| Nanny/maternity matron | -0.02(-0.25, 0.16) | 0.645 |
| **Pre-pregnancy BMI** |  |  |
| Thin(Ref) |  |  |
| Normal | 0.08(-0.01, 0.33) | 0.059 |
| Overweight | 0.05(-0.07, 0.39) | 0.175 |
| Obesity | 0.11(0.10, 0.54) | 0.005 |
| **Suffering from postpartum disease** |  |  |
| No(Ref) |  |  |
| Yes | -0.08(-0.33, -0.06) | 0.005 |
| **Singleton or twin pregnancies** |  |  |
| Singleton pregnancies(Ref) |  |  |
| Twin pregnancies | -0.04(-0.63, 0.13) | 0.189 |
| **Smoking** |  |  |
| Current smoker(Ref) |  |  |
| Ex-smoker | 0.03(-0.61, 1.00) | 0.632 |
| Non-smoker | 0.09(-0.10, 1.22) | 0.097 |
| **Alcohol drinking** |  |  |
| No(Ref) |  |  |
| Yes | -0.05(-0.28, 0.03) | 0.128 |

**Table 4.** After the adjustment of model 3 the association between modes of delivery and vegetables**.**

| Varibles | β (95% CI) | *P*-value |
| --- | --- | --- |
| **Delivery mode** |  |  |
| Vaginal delivery (Ref) |  |  |
| Cesarean section | 0.02(-0.52, 1.36) | 0.381 |
| **Age** |  |  |
| 20-34(Ref) |  |  |
| ≥35 | -0.01(-1.33, 0.96) | 0.754 |
| **Residence** |  |  |
| Urban(Ref) |  |  |
| Rural | -0.03(-2.36, 0.80) | 0.334 |
| **Educational level** |  |  |
| Secondary education and below(Ref) |  |  |
| Tertiary education | 0.09(0.98, 3.45) | <0.001 |
| **Monthly Household income** |  |  |
| ≤4500(Ref) |  |  |
| 4501-9000 | 0.05(-0.24, 2.04) | 0.12 |
| >9000 | 0.08(0.39, 2.65) | 0.008 |
| **Dietary caregivers** |  |  |
| Themselve/husband(Ref) |  |  |
| Parents/Parents-in-law | 0.04(-0.49, 2.11) | 0.221 |
| Nanny/maternity matron | 0.22(3.07, 6.06) | <0.001 |
| **Pre-pregnancy BMI** |  |  |
| Thin(Ref) |  |  |
| Normal | -0.02(-1.68, 0.86) | 0.529 |
| Overweight | 0.03(-1.05, 2.44) | 0.437 |
| Obesity | -0.02(-2.16, 1.14) | 0.544 |
| **Suffering from postpartum disease** |  |  |
| No(Ref) |  |  |
| Yes | -0.02(-1.45, 0.55) | 0.381 |
| **Singleton or twin pregnancies** |  |  |
| Singleton pregnancies(Ref) |  |  |
| Twin pregnancies | 0.01(-2.67, 2.91) | 0.933 |
| **Smoking** |  |  |
| Current smoker(Ref) |  |  |
| Ex-smoker | -0.08(-12.05, 0.38) | 0.066 |
| Non-smoker | -0.07(-9.23, 0.98) | 0.113 |
| **Alcohol drinking** |  |  |
| No(Ref) |  |  |
| Yes | 0.03(0.10, 2.44) | 0.033 |

**Table 5.** After the adjustment of model 3 the association between modes of delivery and fruits.

| Varibles | β (95% CI) | *P*-value |
| --- | --- | --- |
| **Delivery mode** |  |  |
| Vaginal delivery (Ref) |  |  |
| Cesarean section | -0.02(-1.36, 0.62) | 0.464 |
| **Age** |  |  |
| 20-34(Ref) |  |  |
| ≥35 | 0.04(-0.33, 2.10) | 0.153 |
| **Residence** |  |  |
| Urban(Ref) |  |  |
| Rural | -0.01(-1.82, 1.54) | 0.869 |
| **Educational level** |  |  |
| Secondary education and below(Ref) |  |  |
| Tertiary education | -0.07(-3.04, -0.43) | 0.009 |
| **Monthly Household income** |  |  |
| ≤4500(Ref) |  |  |
| 4501-9000 | -0.07(-2.41, -0.02) | 0.046 |
| >9000 | -0.01(-1.42, 0.95) | 0.694 |
| **Dietary caregivers** |  |  |
| Themselve/husband(Ref) |  |  |
| Parents/Parents-in-law | -0.01(-1.42, 1.33) | 0.949 |
| Nanny/maternity matron | 0.11(0.61, 3.76) | 0.007 |
| **Pre-pregnancy BMI** |  |  |
| Thin(Ref) |  |  |
| Normal | -0.01(-1.52, 1.14) | 0.776 |
| Overweight | 0.11(-1.54, 2.15) | 0.745 |
| Obesity | -0.01(-1.84, 1.60) | 0.893 |
| **Suffering from postpartum disease** |  |  |
| No(Ref) |  |  |
| Yes | -0.05(-2.06, 0.04) | 0.060 |
| **Singleton or twin pregnancies** |  |  |
| Singleton pregnancies(Ref) |  |  |
| Twin pregnancies | -0.01(-2.94, 2.86) | 0.979 |
| **Smoking** |  |  |
| Current smoker(Ref) |  |  |
| Ex-smoker | -0.02 (-7.72, 5.16) | 0.697 |
| Non-smoker | 0.01(-4.47, 6.12) | 0.760 |
| **Alcohol drinking** |  |  |
| No(Ref) |  |  |
| Yes | -0.04(-2.06, 0.39) | 0.182 |

**Table 6.** After the adjustment of model 3 the association between modes of delivery and coffee.

| Varibles | β (95% CI) | *P*-value |
| --- | --- | --- |
| **Delivery mode** |  |  |
| Vaginal delivery (Ref) |  |  |
| Cesarean section | -0.05(-3.57, 0.32) | 0.101 |
| **Age** |  |  |
| 20-34(Ref) |  |  |
| ≥35 | 0.01(-1.82, 2.94) | 0.645 |
| **Residence** |  |  |
| Urban(Ref) |  |  |
| Rural | -0.01(-3.85, 2.76) | 0.747 |
| **Educational level** |  |  |
| Secondary education and below(Ref) |  |  |
| Tertiary education | -0.11(-7.62, -2.45) | <0.001 |
| **Monthly Household income** |  |  |
| ≤4500(Ref) |  |  |
| 4501-9000 | -0.07(-4.84, -0.11) | 0.040 |
| >9000 | -0.11(-6.05, -1.39) | 0.002 |
| **Dietary caregivers** |  |  |
| Themselve/husband(Ref) |  |  |
| Parents/Parents-in-law | -0.02(-3.36, 2.06) | 0.638 |
| Nanny/maternity matron | 0.03(-2.09, 4.11) | 0.523 |
| **Pre-pregnancy BMI** |  |  |
| Thin(Ref) |  |  |
| Normal | -0.02(-3.41, 1.77) | 0.533 |
| Overweight | 0.01(-3.20, 3.86) | 0.855 |
| Obesity | -0.04(-5.38, 1.37) | 0.244 |
| **Suffering from postpartum disease** |  |  |
| No(Ref) |  |  |
| Yes | -0.01(-2.52, 1.62) | 0.670 |
| **Singleton or twin pregnancies** |  |  |
| Singleton pregnancies(Ref) |  |  |
| Twin pregnancies | (-7.45, 3.92) | 0.543 |
| **Smoking** |  |  |
| Current smoker(Ref) |  |  |
| Ex-smoker | (-20.98, 4.28) | 0.195 |
| Non-smoker | (-16.75, 4.19) | 0.24 |
| **Alcohol drinking** |  |  |
| No(Ref) |  |  |
| Yes | (-3.36, 1.45) | 0.437 |

**Table 7.** Food grouping for factor analysis.

| Food group | Food items |
| --- | --- |
| Vegetables | raw and cooked vegetables and beans |
| Fruits | fruit and fruit compote |
| Fish | all types ocean and fresh water |
| Fishery products | shrimp and crab or other sea foods |
| Red meat | beef, Lamb, mutton and other non-processed meats |
| Poultry | chicken, duck, goose, pigeon, quail |
| Processed meat | sausage, ham, luncheon meat |
| Milk and buttermilk | plain milk (full-fat, semi-skimmed or skimmed) and plain sour milk |
| Dairy desserts | yogurt (full-fat, semi-skimmed or skimmed yoghurt), custard,  pudding and cream |
| Eggs | fried or baked potatoes and French fries |
| Sugary drinks | sugar-sweetened beverages e.g. soft drinks, fruit-flavored drinks, milk-flavored drinks, sugared waters and commercial teas, sports or energy drinks |
| Nonsugary drinks | water, plain or carbonated with nothing added |
| Tea | tea |
| Coffee | coffee with, without sugar and/or milk |
| Wine | grape wine, yellow rice wine, rice wine, beer |
| Rice | cooked rice and rice flour |
| Wheat | white bread, steamed bread, noodles, dumplings |
| Coarse cereals | corn, millet, sorghum, barley, rye |
| Tubers | potato, taro, yam |
| Whole grains | whole wheat breads, buckwheat |
| Cakes and confectionery | cakes, pancakes, wafels, pastry, ice cream, candy and chocolate |
